# Financial toxicity and the economic burden of Chagas disease: a scoping review

**DOI:** 10.1590/0037-8682-0233-2026

**Published:** 2026-10-16

**Authors:** Douglas de Araújo Costa, Isaque Lima de Farias, Marcos Manoel Sousa Silva, Jhamyle de Oliveira Rodrigues, Kaylane Morais Ribeiro, Anny Karolyne Almeida Oliveira, Mariana Araújo Rios, Beatriz Amorim Beltrão, Paula Sacha Frota Nogueira, Luciana de Alcantara Nogueira

**Affiliations:** 1 Escola de Saúde Pública do Ceará, Fortaleza, CE, Brasil.; 2 Universidade Federal do Ceará, Programa de Pós-Graduação Stricto Sensu em Enfermagem, Fortaleza, CE, Brasil.; 3 Universidade Federal do Ceará, Curso de Graduação em Enfermagem, Fortaleza, CE, Brasil.; 4 Universidade Federal do Ceará, Hospital Universitário Walter Cantídio, Fortaleza, CE, Brasil.; 5 Universidade Federal do Ceará, Departamento de Enfermagem, Fortaleza, CE, Brasil.; 6 Universidade Federal do Paraná, Departamento de Enfermagem, Curitiba, PR, Brasil.

**Keywords:** Health Expenditures, Chagas disease, Financial toxicity, Health equity, Neglected diseases, Social determinants of health

## Abstract

This scoping review mapped, synthesized, and critically analyzed the evidence on financial toxicity in Chagas disease, focusing on its determinants, impacts, and management strategies. Financial toxicity refers to the financial burden and distress experienced by individuals and their families as a result of healthcare expenses. The review followed the Joanna Briggs Institute methodology and the PRISMA-ScR guidelines. Comprehensive searches were conducted across seven electronic databases and the gray literature. Studies were selected and data extracted independently using the population-exposure-outcome framework, and 14 studies included. Most studies described indicators of the economic burden of Chagas disease rather than directly measuring patient-level financial toxicity. Substantial indirect costs were associated with absenteeism, productivity loss, and premature mortality, accounting for 44% to 75% of total costs. The findings also indicated that financial hardship may be intensified by structural vulnerabilities, including extreme poverty, lack of health insurance, and precarious migration status. Available evidence suggests that individuals affected by Chagas disease are at increased risk of healthcare-related financial hardship. However, studies directly assessing financial toxicity are scarce, with most relying on indirect economic indicators. This highlights an important gap in the literature and underscores the need for validated measures to evaluate financial toxicity in this population. Interventions that reduce financial barriers and strengthen access to healthcare services may help mitigate the economic consequences of Chagas disease. Future research should prioritize patient-centered assessments of financial toxicity to better inform policies and strategies aimed at reducing the financial burden associated with the disease.

## INTRODUCTION

Financial toxicity refers to the adverse financial consequences related to healthcare expenses experienced by patients and their households, which may compromise their well-being and reduce quality of life. It is a patient- and household-level construct and should be distinguished from the broader economic burden of disease, which encompasses costs borne by healthcare systems, society, and the economy. This challenge requires coordinated initiatives among professionals, health services, insurers, and governments, that are supported by research identifying feasible interventions^1^. The term encompasses two main components: objective financial burden and subjective financial distress. The construct is structured into three domains: (i) material conditions (e.g., the use of active and passive financial resources), (ii) psychological response (e.g., concerns and distress regarding financial circumstances), and (iii) coping behaviors (actions aimed at managing increased expenses)^2^ ^,^ ^3^.

Chagas disease has economic implications, affecting not only patients and their families, but also health systems and society. Furthermore, because it primarily affects individuals in vulnerable situations, limitations in specialized care access and geographic and socioeconomic barriers may intensify the difficulties, reinforcing cycles of inequality. Regarding the long-term financial impact, this phenomenon is exacerbated by the chronic and progressive nature of the disease, especially clinical complications such as chronic cardiomyopathy, which may require continuous medical follow-up, in addition to specialized care or advanced interventions, including the use of cardiac devices or transplantation. Digestive complications may also require specialized treatments. These expanded healthcare needs can therefore increase direct medical costs along with indirect costs associated with transportation, loss of functional productivity, and barriers to accessing specialized services. A recent systematic review revealed that annual hospital expenditures per patient vary widely, ranging from US$25.47 to US$18,823.74 in purchasing power parity^4^. Because these data are estimates that primarily reflect the economic burden of Chagas disease, including healthcare-related costs, they should not be interpreted as direct measures of financial toxicity. However, they may represent indirect indicators of the potential financial impact that patients and families experience, particularly in contexts in which out-of-pocket expenses and barriers to healthcare access are significant. Thus, understanding how financial toxicity manifests in patients affected by Chagas disease is critical.

Financial toxicity has been extensively debated in oncology, often with focus placed on high-cost technological innovations^2^ however, limited literature is present on tropical diseases. Although previous studies have investigated the economic burden of Chagas disease, including healthcare costs and productivity losses, evidence synthesizing how these economic consequences translate into patient- and household-level financial toxicity is still limited. Gaps remain regarding financial distress, out-of-pocket expenditures, hidden costs, access barriers, and coping behaviors among individuals affected by Chagas disease, thus limiting our understanding. We conducted a scoping review, suited for mapping literature on evolving or emerging topics and identifying research gaps^1^ ^,^ ^4^ ^,^ ^5^.

The objectives were to map, categorize, and synthesize the available evidence on financial toxicity among patients with Chagas disease, considering its determinants, patient-level impacts, and management strategies reported in the scientific and gray literature. The primary research question was: “What evidence is available on financial toxicity in patients with Chagas disease?” The secondary questions were: “What factors contribute to financial toxicity in patients with Chagas disease?” and “What are the outcomes of financial toxicity in patients with Chagas disease?”

## METHODS

### Protocol

This scoping review was conducted in accordance with the methodological recommendations of the Joanna Briggs Institute Manual for Evidence Synthesis^6^ and Preferred Reporting Items for Systematic Reviews and Meta-Analyses extension for Scoping Reviews (PRISMA-ScR) guidelines (Supplementary Material 1)^7^. The review protocol was registered in the Open Science Framework on February 28, 2025 (https://doi.org/10.17605/OSF.IO/M3FER).

### Eligibility criteria and search strategy

Study inclusion was defined by the population-exposure-outcome (PEO) framework^8^ and specific evidence source types, because based on the research questions, the aim was to investigate how exposure to financial toxicity and economic burden of Chagas disease impacts individuals’ lives. Therefore, the PEO framework enabled us to organize the main aspects of the investigation in a clearer and structured manner. The review included individuals affected by Chagas disease across different clinical stages, including acute infection, chronic indeterminate disease, chronic Chagas cardiomyopathy, digestive forms, and advanced disease stages. Studies involving family members or caregivers and mortality-related outcomes were also considered when relevant to financial toxicity and socioeconomic impacts. The exposure of interest was financial toxicity among individuals affected by Chagas disease. For this review, financial toxicity was defined as the patient- and family-level financial consequences related to the disease, including direct expenses, financial difficulties, income loss, coping strategies, and material consequences. Healthcare system costs, productivity losses, and social vulnerability factors were interpreted as indirect evidence of the economic burden of Chagas disease. Eligible outcomes were financial well-being, quality of life, mental health outcomes (e.g., depression and anxiety), welfare benefits, counseling services, and any aggravating or mitigating factors or barriers related to financial toxicity. Eligible evidence sources included primary clinical studies employing quantitative, qualitative, or mixed-methods designs from both the peer-reviewed and gray literature; review protocols, literature reviews of any type, conference abstracts, editorials, expert opinions, and other non-peer-reviewed documents were excluded. No restrictions were placed on country or study setting. Given the geographic distribution of Chagas disease, studies published in English, Portuguese, and Spanish were considered eligible.

The database search was conducted in two phases: peer-reviewed followed by gray literature. For the peer-reviewed literature, searches were conducted in March 2025 across PubMed, Scopus, Embase, Cochrane Library, the Web of Science platform, BDENF (a database for the nursing field), and LILACS (Latin American and Caribbean Health Sciences Literature). For the gray literature, searches were conducted between March and April 2025 using Science.gov, the CAPES Theses and Dissertations Catalog, Google Scholar, WorldCat, and the National ETD Portal (a portal providing access to electronic theses and dissertations). In both phases, searches were performed independently by four investigators.

The search strategies were constructed using keywords identified in preliminary records alongside standardized medical terminologies, including Medical Subject Headings, the Embase (the Embase thesaurus) and DeCS Health Sciences Descriptors. Terms were combined using Boolean operators (AND, OR) and truncation to maximize search sensitivity. Although both search phases adhered to these foundational criteria, the specific strategies applied to the gray literature were adapted to the unique syntax requirements of each database. An updated search was conducted in March to July 2025 and no additional eligible studies were identified. Full details of the search strategies are provided in Supplementary Material 2.

### Screening and data extraction

All peer-reviewed literature retrieved through the search strategies was exported to Rayyan^9^ for screening, whereas the gray literature records were managed separately and compiled in a Microsoft Excel spreadsheet. These records underwent the same process of assessment for eligibility, including title and abstract screening, when available, and full-text evaluation according to the previously defined inclusion and exclusion criteria. Duplicate records identified across different gray literature sources were resolved before the screening process. Three reviewers independently performed the initial screening of titles and abstracts against the eligibility criteria. Eligibility decisions were independently assessed by the reviewers, and any disagreements were resolved through discussion and consensus. Each investigator was responsible for searching a specific database. To ensure consistency, all investigators followed the same search strategy and applied the same eligibility criteria. Subsequently, all investigators reviewed the results. The full text of potentially eligible studies were then retrieved and assessed independently by the same three reviewers to determine final inclusion. Any discrepancies during the selection process were resolved through consensus among the three reviewers. The study selection process, including reasons for exclusion, is illustrated in the PRISMA-ScR flow diagram (**Figure 1**)^10^.

Data were extracted such that study identifiers, study designs, conceptualizations of financial toxicity, study population, and primary findings were captured. The data extraction template is available in Supplementary Material 3.

### Data synthesis

The included studies were characterized using a standardized table summarizing the first author, year of publication, country of origin, study design, the specific dimension of financial toxicity evaluated, and the study population. The categories used for synthesis were defined a priori based on the conceptual framework of financial toxicity and the objectives of this review. The extracted evidence was organized into domains related to patient- and household-level financial consequences, broader indicators of economic burden, direct and indirect contributing factors, barriers to healthcare access, and impacts on quality of life. Additional subcategories were refined during the synthesis process according to patterns identified across the included studies. Given the methodological and conceptual heterogeneity of the included studies, no statistical pooling or meta-analysis was performed. The findings were synthesized descriptively and narratively to characterize the extent, nature, and distribution of the available evidence. The findings were synthesized in both tabular and narrative forms.

### Ethical considerations

Because this scoping review relied exclusively on the secondary analysis and synthesis of publicly available, deidentified data from published scientific literature, no direct human-subject intervention was conducted, and no identifiable personal data accessed. Consequently, the study was exempt from review or approval by an institutional review board or research ethics committee.

## RESULTS

### Selection of sources of evidence

In total, 554 citations were initially identified across the databases searched (Supplementary Material 2). After eliminating 70 duplicate entries, the titles and abstracts of 484 unique records were screened, leading to 460 articles being excluded. The remaining 24 articles underwent a thorough full-text evaluation, of which 14 were excluded because they did not meet the eligibility criteria; the specific justifications for these exclusions are provided in Supplementary Material 4.

Consequently, 10 studies were initially selected. A subsequent backward citation search of these 10 articles uncovered 12 extra references. After evaluating the full text of these additional articles, four met the criteria and were incorporated into the review (note that the reasons for exclusion in this supplementary search are not listed in Supplementary Material 4). Ultimately, the final sample comprised 14 studies. The entire study selection workflow is visually detailed in the PRISMA-ScR flow diagram (Figure 1)**.**

**FIGURE 1:**
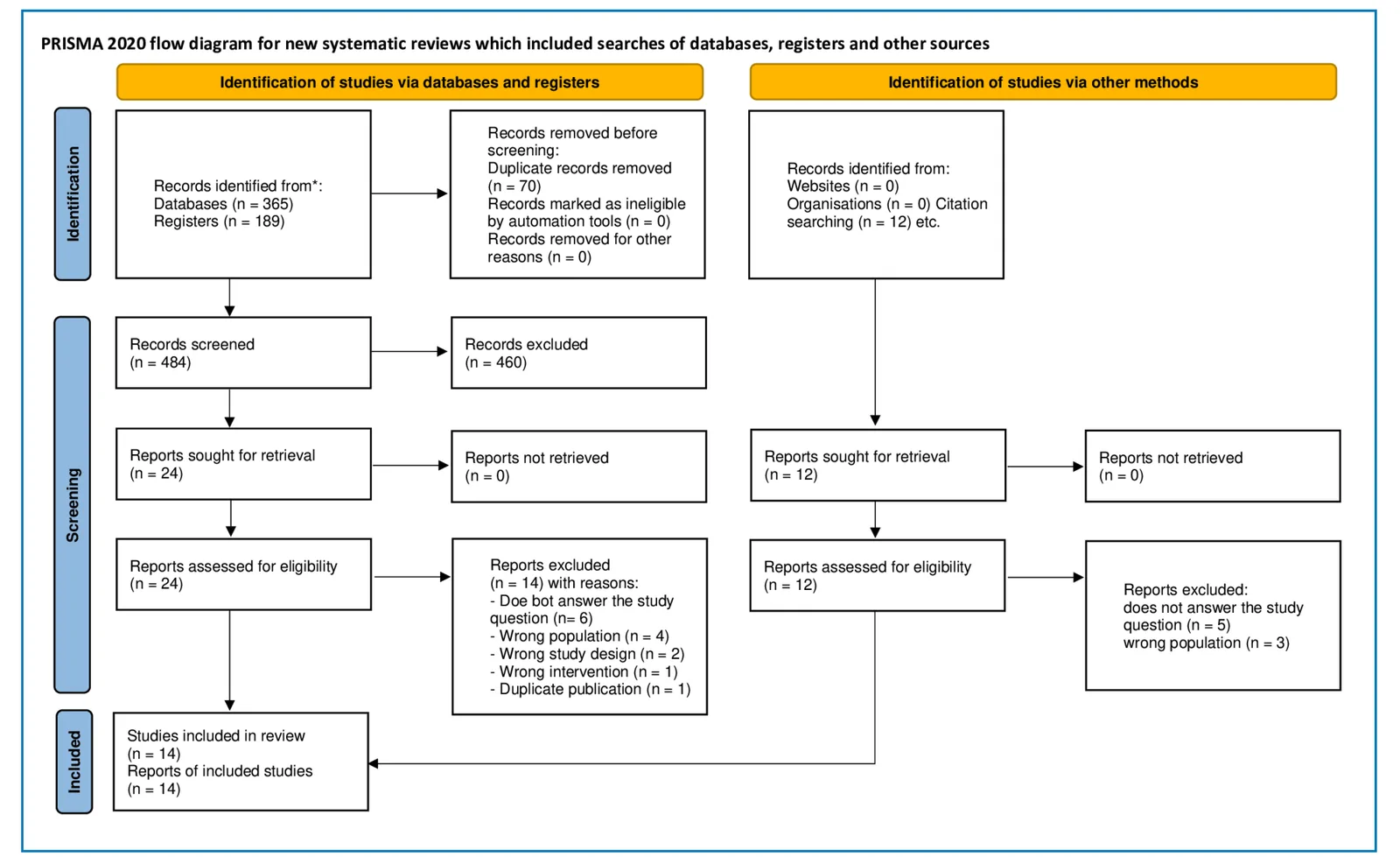
PRISMA-ScR flow diagram illustrating the study selection and screening process¹⁰.

### Characteristics of the included studies

The characteristics of the 14 included studies, published between 2007 and 2023, are summarized in Table 1. Among the methodological designs, cross-sectional studies predominated (n=5)^11^ ^-^ ^14^, followed by epidemiological studies using secondary data (n=3)^15^ ^-^ ^17^, and cohort studies (n=2)^18^ ^,^ ^19^. The remaining designs included one qualitative study^11^, one mixed-methods study^20^, one economic modeling study^21^, and one quantitative observational study^22^. Most of the research was conducted in South America, with the highest number of studies published in Brazil (n=4) and Colombia (n=4). Five studies incorporated comparison groups, such as patients with cardiomyopathy of other etiologies^18^ ^,^ ^19^ or seronegative blood donors^13^, to isolate disease effects. The other studies did not directly involve patients, relying instead on secondary mortality data^16^, funding records^15^, or computational simulation models^21^ ^,^ ^23^. The frequency of studies according to the dimensions of financial toxicity identified in this review is summarized in Table 2.

**TABLE 1:** Characteristics of the 14 included studies.

Author, Year	Country	Study type	Financial toxicity assessment	Population
Parisi et al., 2020	Bolivia	Mixed methods	Socioeconomic and medical data form, including focus group discussions	Adults aged ≥18 residing in the municipality of Monteagudo for at least 6 months prior to the study
Abuhab et al., 2013	Brazil	Prospective cohort	Comparative documentary study of medical records	Adults with Chagasic (58) and non-Chagasic (519) cardiomyopathy admitted to a university cardiology hospital in São Paulo, Brazil
Herazo et al., 2023	Colombia	Cross-sectional	Calculations of medical and nonmedical expenses, and work-related income loss	Patients with a confirmed diagnosis of *Trypanosoma cruzi* infection treated in two endemic municipalities in Colombia
Parra, 2007	Brazil	Retrospective cohort	Socioeconomic documentary evaluation	Patients undergoing socioeconomic evaluation prior to heart transplantation at the Hospital de Base de São José do Rio Preto
Sangenito, Branquinha, & Santos, (2020)	Brazil	Epidemiological, using secondary data	Evaluation of the impact of financial investments in Chagas disease compared to other neglected diseases	Annual funding data for neglected diseases provided by government, private, and philanthropic institutions
Jackson et al., 2012	Switzerland	Cross-sectional	Interviews using an instrument for demographic, behavioral, socioeconomic, and mental health data in patients with Chagas disease	Latin American migrants aged >15 with Chagas disease at the University Hospitals of Geneva, Switzerland
Olivera et al., 2021	Colombia	Epidemiological, using secondary data	Evaluation of mortality data to estimate the cost of premature mortality and potential years of work lost	Deaths from Chagas disease between 2010 and 2017 among men (aged 18-62) or women (aged 18-57) in Colombia
Olivera & Buitrago, 2020	Colombia	Cross-sectional	Estimation of direct medical costs, nonmedical costs, and indirect costs	Direct medical and nonmedical costs and indirect costs associated with patients with Chagas disease
Baldoni et al., 2023	Brazil	Cross-sectional	Calculation of sociodemographic, behavioral, and clinical variables using means, standard deviations, and relative and/or absolute frequencies	Participants from two blood donation centers: São Paulo (capital) and Montes Claros, Minas Gerais, Brazil
Ramsey et al., 2014	Mexico	Economic modeling	Direct costs (medical treatment) and indirect costs (lost work days)	Patients with Chagas disease in Mexico
Fernández et al., 2019	Argentina	Quantitative observational	Association between the disease and barriers to accessing health services	Rural community in the municipality of Pampa del Indio, Argentina.
Santos-Filho et al., 2018	Brazil	Cross-sectional	Collection of sociodemographic data, household resident data, and per capita income	Outpatients followed at the Evandro Chagas National Institute of Infectious Diseases, Brazil
Olivera et al., 2018	Colombia	Qualitative	Interviews with managers and health professionals on diagnostic pathways for Chagas disease and barriers faced by patients and health services	Key informants including health policymakers, department heads, hospital directors, and primary care physicians with expertise in treating Chagas disease
Lee et al., 2013	United States	Quantitative observational study	Computer simulation to analyze treatment-seeking probability, costs, duration of absenteeism, and age at initial infection	TreeAge Pro (version 2009) and Microsoft Excel (version 2007) used to develop a Markov computer simulation model

**TABLE 2:** Frequency of studies according to the dimensions of financial toxicity identified in Chagas disease.

Dimensions of financial toxicity	Frequency	Author, Year
Direct costs	7	Herazo et al., 2023; Abuhab et al., 2013; Olivera & Buitrago, 2019; Ramsey et al., 2014; Lee et al., 2013; Olivera, Palencia-Sánchez, & Riaño-Casallas, 2021; Sangenito et al., 2020
Indirect costs	6	Herazo et al., 2023; Olivera & Buitrago, 2019; Olivera et al., 2021; Ramsey et al., 2014; Lee et al., 2013; Abuhab et al., 2013
Socioeconomic vulnerability	3	Jackson et al., 2012; Santos-Filho et al., 2018; Parra, 2007
Barriers to accessing healthcare	3	Parisi et al., 2020; Olivera et al., 2018; Fernández, Gaspe, & Gürtler, 2019
Quality of life	3	Jackson et al., 2012; Baldoni et al., 2023; Santos-Filho et al., 2018
Macroeconomic burden	5	Lee et al., 2013; Olivera & Buitrago, 2019; Olivera, Palencia-Sánchez, & Riaño-Casallas, 2021; Sangenito, Branquinha, & Santos, 2020; Abuhab et al., 2013

Diverse methodological procedures were used to analyze financial toxicity. Because most studies did not directly measure patient-level financial toxicity, they assessed related outcomes, such as overall economic burden, hospital costs, and productivity loss. Therefore, these findings serve as direct evidence, indirect proxies, contextual determinants, or macroeconomic evidence. In five studies, data were collected on direct and indirect costs through questionnaires and interviews^11^ ^,^ ^12^ ^,^ ^14^ ^,^ ^20^ ^,^ ^22^. Secondary data analysis, using data from medical records and national registries, was performed in three studies to estimate economic impact^16^ ^-^ ^18^. The World Health Organization - Quality of Life - Brief (WHOQOL-BREF) questionnaire was used in three studies to assess quality of life^12^ ^-^ ^14^ simulation models were used in two to project disease costs^21^ ^,^ ^23^. Reported outcomes included the quantification of direct (e.g., transportation and food expenses)^11^ ^,^ ^17^ and indirect (e.g., productivity loss)^16^ ^,^ ^21^ ^,^ ^23^ costs. An association between the disease and barriers to accessing health services was also reported^20^ ^,^ ^22^ ^,^ ^24^. In the studies that used the WHOQOL-BREF tool, scores in the physical and psychological domains were specifically reported for patients with Chagas disease^13^ ^,^ ^14^. In one study, data concerning global funding directed toward Chagas disease were described^15^.

Questionnaires, interviews, and self-reported data specifically provided information on sociodemographic characteristics, direct and indirect costs, income losses, and health perceptions^11^ ^,^ ^12^ ^,^ ^14^. Socioeconomic and vulnerability indicators, including socioeconomic status, asset index, and access to healthcare and sanitation, were also factored into the analyses^20^ ^,^ ^22^.

Instead of directly quantifying patient-level financial toxicity, the studies measured broader health-system and societal burdens. Hospitalization and total disease costs were used to estimate the system’s burden, whereas direct and indirect expenses served as indirect proxies for household hardship. Mortality-related productivity losses provided macroeconomic evidence^15^ ^-^ ^18^ ^,^ ^21^. In one study, simulation models were used to estimate age at onset, duration of chronic disease, and projections of disease burden, with validation performed against previous findings^23^. Another study relied on documentary analysis and secondary records, such as official reports and institutional databases^24^.

Regarding prognosis and clinical outcomes, the survival probability of patients with Chagas disease on the heart transplant waiting list was compared with that of patients without Chagas disease^19^. Quality of life was assessed using scales converted to linear values, and between-group comparisons were made using logistic regression models and advanced statistical analyses^13^.

### Evidence of financial toxicity related to Chagas disease

The synthesized evidence (Table 3 and Table 4) spans several interconnected domains. Regarding direct costs, five studies^11^ ^,^ ^12^ ^,^ ^14^ ^,^ ^20^ ^,^ ^22^ assessed patients’ out-of-pocket and nonmedical expenses (e.g., transportation) via questionnaires, whereas seven^13^ ^,^ ^15^ ^,^ ^16^ ^,^ ^18^ ^,^ ^21^ ^,^ ^23^ ^,^ ^24^ used secondary data or modeling to estimate health-system medical expenditures such as hospitalizations. In six studies^15^ ^-^ ^18^ ^,^ ^21^ ^,^ ^23^, indirect costs and productivity loss due to absenteeism or premature mortality were evaluated. Access barriers were explicitly addressed in three studies^20^ ^,^ ^22^ ^,^ ^24^ and social vulnerability was identified as a key contextual determinant exacerbating financial distress in three others^12^ ^,^ ^20^ ^,^ ^22^. In three studies^12^ ^-^ ^14^, impacts on quality of life were evaluated using standardized instruments; in five studies^15^ ^-^ ^17^ ^,^ ^21^ ^,^ ^23^, the broader macroeconomic burden of the disease on society was estimated.

**TABLE 3:** General evidence of financial toxicity in Chagas disease.

Author, Year	Evidence of financial toxicity
Parisi et al., 2020	Associated extreme poverty with Chagas disease, hindering access to care. Poverty led to the prioritization of urgent needs (work, food) over health.
Abuhab et al., 2013	Patients with Chagasic cardiomyopathy had higher total hospital costs than patients with non-Chagasic cardiomyopathy, primarily driven by higher intensive care costs.
Herazo et al., 2023	Care at specialized reference hospitals generated significantly higher direct costs (transportation, food, lodging) and indirect costs (income loss) for patients compared to primary care.
Parra, 2007	Described the socioeconomic vulnerability of patients on the heart transplant waiting list: the majority (87%) belonged to a low social class, and had limited education and a median monthly income of 2.5 minimum wages.
Sangenito et al., 2020	Provided a macro-level perspective on funding in Chagas disease, which was disproportionately low at US$236.3 million (0.67% of the total budget for neglected diseases) relative to its economic impact of over US$7 billion annually (2009-2018).
Jackson et al., 2012	Highlighted the financial burden on immigrants: high poverty rates (89.1%), lack of health insurance (72.3%), and precarious migratory status (83.3%) were barriers to gaining access to the healthcare system.
Olivera et al., 2021	Quantified the cost of lost productivity due to premature mortality (years of life lost), estimated at US$29.6 million from 2010 to 2017.
Olivera and Buitrago, 2020	Detailed Chagas disease costs in Colombia (2017), categorized as: direct medical costs (US$3 million), direct nonmedical costs (transportation, food-$1.4 million), and indirect costs (productivity loss- US$5.8 million).
Baldoni et al., 2023	Patients with Chagas disease exhibited significantly lower mean monthly income (US$22.97 vs. US$32.07) and lower educational levels, which were factors associated with lower quality of life scores.
Ramsey et al., 2014	Productivity loss (indirect costs) was the primary source of financial toxicity (44%-75% of costs). Early diagnosis and treatment reduced total lifetime costs (US$10,160) compared to no treatment (US$11,877).
Fernández et al., 2019	Described the socioeconomic vulnerability of rural communities in the Argentine Chaco, which exhibited poorer housing conditions, lower educational levels, and higher dependence on social support compared to other groups.
Santos-Filho et al., 2018	Demonstrated that low per capita income was associated with lower scores in physical, psychological, and environmental domains, as well as overall quality of life.
Olivera et al., 2018	Identified financial barriers to access to diagnosis, with patients incurring costs for confirmatory tests (~US$25), copayments (~US$1- US$8), and transportation to health centers and laboratories.
Lee et al., 2013	Estimated the global economic burden of Chagas disease at US$7.19 billion per year. The lifetime cost per infected person was US$3,456 (healthcare costs). Productivity loss from mortality was a substantial component of this burden.

**TABLE 4:** Types of evidence of financial toxicity in Chagas disease.

Type	Category	Meaning	Evidence
Direct costs (medical and nonmedical)	Health system and Patient/Household	Direct costs refer to patients’ financial expenditures related to the healthcare system and logistical needs. In countries with universal public health systems, diagnosis or etiological treatment may be formally available, but patients still face substantial logistical costs (transportation, food, lodging) and fragmented referral pathways.	(i) Hospitalization and complication costs: Patients with Chagasic cardiomyopathy incur higher total hospitalization costs compared with patients with other cardiomyopathies, mainly due to a greater need for intensive care^12^. (ii) Logistical costs (transportation, accommodation, food): Care provided in referral (specialized) hospitals generates significantly higher transportation, food, and accommodation costs for patients compared with primary care^14^. Transportation is identified as a major component of the nonmedical costs^18^. (iii) Access and copayment costs: Even with theoretical coverage, patients face financial barriers, such as the need to pay for confirmatory tests (e.g., approximately US$25 in Colombia) and copayments for consultations and diagnostic tests^23^. (iv) Components of medical costs: The main drivers of direct medical costs include cardiac device implantation, ventricular function tests, and myocardial perfusion testing^18^.
Indirect costs (productivity loss)	Patient/Household	Indirect costs relate to the patient’s personal loss of work capacity and lost workdays.	(i) Income loss (absenteeism): Patients report significant income loss (lost workdays) to attend medical appointments, with greater losses experienced when seeking care in specialized hospitals^13^. (ii) Main component of economic burden: Productivity loss (including presenteeism, absenteeism, and mortality) accounts for the largest proportion of total disease costs^18^ ^,^ ^20^ ^.^ ^24^. In one model, indirect costs accounted for 44% of the total costs in treatment scenarios and for up to 75% in scenarios without diagnosis^20^. (iii) Premature mortality: Productivity loss due to premature mortality (potential years of working life lost) represents a substantial economic cost^18^.
Access barriers and socioeconomic vulnerability	Contextual	Financial toxicity is intrinsically linked to patients’ socioeconomic vulnerability.	(i) Poverty and competing priorities: Extreme poverty, associated with Chagas disease^11^ ^,^ ^16^, forces patients to prioritize urgent needs (work, food) over healthcare^11^. (ii) Lack of insurance and migration status: Lack of health insurance and precarious migration status^16^ are critical barriers that prevent access to the healthcare system. (iii) Socioeconomic determinants: Patients with Chagas disease have, on average, lower monthly income, lower educational and literacy levels^19^, poorer housing conditions^14^ ^,^ ^21^ ^,^ and greater dependence on social support^21^, exacerbating financial vulnerability.
Impact on quality of life	Patient/Household	Financial costs have a direct impact on patient well-being and are a significant burden to society.	(i) Quality of life: Low per capita income is directly associated with poorer scores in the physical, psychological, environmental, and overall quality of life domains^22^. Patients with Chagas disease present with significantly lower quality of life in the physical and psychological domains^19^. (ii) Global economic burden: The annual global cost of Chagas disease is estimated at US$7.19 billion, with a lifetime cost per infected person estimated at US$3,456 (healthcare costs) or up to US$27,684 (including disability-adjusted life years)^24^. (iii) Cost-effectiveness of treatment: Early diagnosis and treatment have the lowest lifetime cost (US$10,160), whereas the absence of diagnosis and/or treatment increases costs (US$11,877), demonstrating that early treatment reduces long-term financial toxicity^20^.

## DISCUSSION

The aim of this scoping review was to map, categorize and synthesize the literature on financial toxicity in Chagas disease. Although financial security was explored as a conceptual framework for this review, none of the studies included assessment using specific instruments or structures. The evidence identified is reproduced in widely recognized dimensions such as direct and indirect costs related to illness, socioeconomic vulnerability, barriers to access to health services, and impacts on quality of life. Evidence shows that direct medical costs are distributed across multiple levels: health systems face the macroeconomic burden of complex chronic-phase hospitalizations^4^ ^,^ ^25^, while patients and households bear significant out-of-pocket expenses. Consequently, the average annual hospitalization cost per patient varies substantially, ranging from approximately US$25 to over US$18,000, depending on symptom severity and complexity^4^ ^,^ ^26^.

The most frequent complications requiring hospitalization included cardiomyopathies, megaesophagus, and megacolon^27^ ^-^ ^29^, which require costly surgical interventions and intensive care, with lifetime costs >US$14,000^4^ ^,^ ^28^. However, distinguishing the broader economic burden of Chagas disease from patient- and household-level financial consequences is important. Although the direct medical costs of diagnosis and treatment contribute substantially to the overall economic burden of the disease, access to appropriate care remains challenging in many settings. Patients and households may therefore face financial burdens related to out-of-pocket expenditures, nonmedical expenses, and indirect costs associated with seeking and receiving care^30^ ^-^ ^33^.

Several primary drivers of medical expenditures have been identified: diagnostic testing, specialized consultations, digestive and cardiac surgeries, pacemaker and implantable cardioverter-defibrillator implantation, heart transplantation, and pharmacological therapy^4^ ^,^ ^27^ ^,^ ^30^. Notably, a randomized clinical trial demonstrated that advanced care technologies, specifically the use of implantable cardioverter-defibrillators over amiodarone, reduced sudden cardiac death by 72% and heart failure-related hospitalizations by 47%^34^. However, inferring long-term economic benefits from these clinical outcomes requires caution. Reduced hospitalizations do not necessarily equate to less financial toxicity or economic burden unless supported by formal cost-effectiveness analyses.

This review’s findings also underscore that direct costs extend beyond clinical expenses, because expenditures were heavily influenced by logistical and systemic barriers. Systematic evidence indicated that nonmedical expenses-specifically transportation, food, and accommodation-constitute a significant burden, particularly when care was centralized in referral centers^4^ ^,^ ^31^ ^,^ ^35^. Such centralization exacerbates financial toxicity by creating geographic barriers that often lead to reduced therapeutic adherence and compromised clinical follow-up.

Health systems vary significantly across South America, preventing a homogeneous analysis. In Brazil and Colombia, where the public system provides diagnosis and etiological treatment (e.g., benznidazole), financial toxicity rarely stems from these specific interventions. Instead, impoverished families, who typically lack private insurance^4^ ^,^ ^36^, face out-of-pocket expenses primarily from logistical burdens (transportation, food, lodging), lost workdays due to referral delays, and long-term follow-up costs. However, systemic limitations to healthcare access still lead to occasional copayments or self-provision of other medications, perpetuating inequalities^32^ ^,^ ^36^ ^,^ ^37^.

Beyond direct medical and logistical costs, the economic burden of Chagas disease is heavily influenced by indirect factors, which must be clearly distinguished into patient-level income loss and societal productivity loss. At the household level, financial toxicity is significantly driven by work absenteeism, which directly impacts family income and reinforces cycles of inequality^36^. In a Brazilian study, absenteeism accounted for 28% of the average indirect expenditures over the lifetime^33^. The frequent loss of workdays to attend centralized appointments creates a major economic barrier for patients^38^, emphasizing the urgent need to decentralize care to primary health services to mitigate prolonged work absences.

Conversely, productivity loss due to premature mortality represents a broader macroeconomic burden rather than direct patient-level financial toxicity. National and global studies have demonstrated that this societal loss can exceed direct medical costs^37^. In Colombia, for example, premature mortality led to macroeconomic productivity losses estimated at approximately US$29.6 million (US$29,683,913) over 7 years, with over 48,000 potential years of work lost^16^. Although not a direct measure of household financial hardship, these findings provide a quantitative measure of the immense societal impact the disease has and highlight the need for public policies that prioritize resource allocation to early detection, treatment, and rehabilitation^16^ ^,^ ^39^.

The statement that a 2025 study showed a distribution of direct care costs (72%) and indirect productivity losses (28%) should be contextualized. Whether this study was included in the scoping review or used only as external contextual literature is unclear^37^. The authors should clearly distinguish between findings derived from included sources and those included from external studies that were only cited to support the discussion.

Descriptive analyses of Chagas disease notifications and vulnerability indicators suggest that unplanned urban growth and the expansion of informal settlements directly influence public health outcomes, creating vulnerabilities that facilitate disease transmission^40^. Such findings corroborate those of our review, namely, that socioeconomic vulnerability is closely associated with inadequate housing and a heavy reliance on social support and public assistance.

A 2024 study involving Bolivian immigrants reported significant barriers to healthcare access, including financial constraints and delays in obtaining care⁴¹. These obstacles hinder timely intervention, enabling the disease to progress. Similar issues were identified in our review, indicating that high rates of poverty and a lack of health insurance among immigrant populations prevent effective access to quality care^41^. However, clarifying that social vulnerability and barriers to access are not direct measures of financial toxicity is important. Instead, these factors act as critical determinants and contextual drivers that exacerbate the financial burden.

According to Silva et al.^42^, patients with Chagas cardiomyopathy face severe social challenges undermining quality of life. Their multilevel model demonstrated that social, community, and individual factors negatively affect both physical and psychological health. Our findings align with this perspective, suggesting that factors such as low per capita income are closely linked to poorer patient outcomes. However, although impaired quality of life is an outcome associated with Chagas disease and socioeconomic vulnerability, it should not be interpreted as direct evidence of financial toxicity unless the financial pathway is explicitly demonstrated.

## LIMITATIONS AND SUGGESTIONS

The construct of financial toxicity remains underexplored in the literature on Chagas disease, often appearing as a secondary theme. This is compounded by marked methodological heterogeneity (lacks a standardized evaluation approach), relying instead on overlapping metrics of direct and indirect costs, access barriers, and quality of life. The review process itself presented with limitations: a potentially outdated search, lack of formal critical appraisal, possible inconsistencies in gray literature eligibility, use of indirect proxies for financial toxicity, and difficulty in comparing monetary estimates across countries and years. Future research should use longitudinal designs to systematically track the evolution of financial toxicity throughout the natural history of Chagas disease because financial burden may relate to diagnosis, access to etiological treatment, long-term monitoring, chronic cardiomyopathy, digestive complications, device therapy, hospitalization, transplantation, disability, and mortality. Furthermore, given the multifaceted nature, investigations must emphasize its impact across diverse economic, ethnic, and social contexts. Included studies cautiously support the plausible implication that structured institutional care pathways are needed to mitigate direct household expenditures and guarantee healthcare access. Meanwhile, broader interpretations based on the contextual literature emphasize the need for comprehensive public policies, such as decentralized care, strengthening primary healthcare, and social protection policies, to promote health equity.

## CONCLUSION

This scoping review found that financial toxicity remains underexplored as a distinct patient- and household-level construct. None of the included studies directly assessed financial toxicity using a specific instrument or standardized framework. Instead, the available evidence primarily described related dimensions, including direct and indirect costs, productivity losses, barriers to healthcare access, socioeconomic vulnerability, and quality-of-life outcomes.

Thus suggesting that Chagas disease may be associated with substantial economic and financial challenges; however, the predominance of indirect indicators and the marked heterogeneity in study designs, populations, and outcome measures limit direct conclusions regarding financial toxicity extent. Future research should prioritize the use of standardized, patient-centered approaches to directly assess financial toxicity and clarify how its manifestations vary across different clinical stages and socioeconomic contexts.

## DECLARATION OF THE USE OF GENERATIVE AI AND AI-ASSISTED TECHNOLOGIES IN THE WRITING PROCESS

During the preparation of this manuscript, the authors used generative artificial intelligence and AI-assisted technologies to support language editing and improve the clarity and readability of the text. The authors critically reviewed and revised all AI-assisted content and take full responsibility for the accuracy, integrity, and originality of the manuscript. No AI system was used to generate, analyze, or interpret the scientific data or to replace the authors’ scientific judgment.

## Data Availability

Research data is available in the body of the document.
