# Supplementary material for: Financial toxicity and the economic burden of Chagas disease: a scoping review
Source: Rev Soc Bras Med Trop. 2026 Oct 16;59:e0233-2026. doi: 10.1590/0037-8682-0233-2026 (PMC13674208; doi:10.1590/0037-8682-0233-2026)
Supplement: Supplementary file 2 [file 1678-9849-rsbmt-59-e0233-2026-md2.pdf]

Supplementary Material 2. Search strategy and results obtained in the different databases

DATABASE	SEARCH CHAIN	FILTERS	NUMBER OF REFERENCES/ ARTICLES RETRIEVED.
Scopus<br><br>***Search conducted on March 6, 2025***	("Trypanosoma cruzi" OR "Chagas Disease" OR "Neglected Tropical Diseases" OR "Vector-Borne Diseases") AND ("Health Expenditures" OR "Poverty" OR "Cost of Illness" OR "Financial Stress" OR "Health Care Costs") AND ("Quality of Life" OR "Health Status" OR "Mental Health" OR "Depression" OR "Anxiety" OR "Stress, Psychological" OR "Social Support" OR "Social Isolation" OR "Health Services Accessibility" OR "Healthcare Disparities" OR "Insurance, Health" OR "Counseling" OR "Housing" OR "Ill-Housed Persons" OR "Living Conditions" OR "Social Determinants of Health" OR "Prejudice" OR "Social Stigma" OR "Social Class" OR "Social Marginalization" OR "Occupational Health" OR "Job Insecurity" OR "Unemployment" OR "Food Supply" OR "Medication Adherence" OR "Delayed Diagnosis" OR "Patient Dropouts" OR "Health Services Needs and Demand" OR "Transportation" OR "Rural Health Services")	Last 10 years	***172***
Medline (PubMed)<br><br>***Search conducted on March 6, 2025***	("Trypanosoma cruzi" OR "Chagas Disease" OR "Neglected Tropical Diseases" OR "Vector-Borne Diseases") AND ("Health Expenditures" OR "Poverty" OR "Cost of Illness" OR "Financial Stress" OR "Health Care Costs") AND ("Quality of Life" OR "Health Status" OR "Mental Health" OR "Depression" OR "Anxiety" OR "Stress, Psychological" OR "Social Support" OR "Social Isolation" OR "Health Services Accessibility" OR "Healthcare Disparities" OR "Insurance, Health" OR "Counseling" OR "Housing" OR "Ill-Housed Persons" OR "Living Conditions" OR "Social Determinants of Health" OR "Prejudice" OR "Social Stigma" OR "Social Class" OR "Social Marginalization" OR "Occupational Health" OR "Job Insecurity" OR "Unemployment" OR "Food Supply" OR "Medication Adherence" OR "Delayed Diagnosis" OR "Patient Dropouts" OR "Health Services Needs and Demand" OR "Transportation" OR "Rural Health Services")	Last 10 years	***86***
Cochrane<br><br>***Search conducted on March 6, 2025***	("Trypanosoma cruzi" OR "Chagas Disease" OR "Neglected Tropical Diseases" OR "Vector-Borne Diseases") AND ("Health Expenditures" OR "Poverty" OR "Cost of Illness" OR "Financial Stress" OR "Health Care Costs") AND ("Quality of Life" OR "Health Status" OR "Mental Health" OR "Depression" OR "Anxiety" OR "Stress, Psychological" OR "Social Support" OR "Social Isolation" OR "Health Services Accessibility" OR "Healthcare Disparities" OR "Insurance, Health" OR "Counseling" OR "Housing" OR "Ill-Housed Persons" OR "Living Conditions" OR "Social Determinants of Health" OR "Prejudice" OR "Social Stigma" OR "Social Class" OR "Social Marginalization" OR "Occupational Health" OR "Job Insecurity" OR "Unemployment" OR "Food Supply" OR "Medication Adherence" OR "Delayed Diagnosis" OR "Patient Dropouts" OR "Health Services Needs and Demand" OR "Transportation" OR "Rural Health Services")	Last 10 years	***5***
Web of Science	("Trypanosoma cruzi" OR "Chagas Disease" OR "Neglected Tropical Diseases" OR "Vector-Borne Diseases") AND ("Health Expenditures" OR "Poverty" OR "Cost of Illness" OR "Financial Stress" OR "Health Care Costs") AND ("Quality of Life" OR "Health Status" OR	Last 10 years	***50***

Search conducted on March 6, 2025	"Mental Health" OR "Depression" OR "Anxiety" OR "Stress, Psychological" OR "Social Support" OR "Social Isolation" OR "Health Services Accessibility" OR "Healthcare Disparities" OR "Insurance, Health" OR "Counseling" OR "Housing" OR "Ill-Housed Persons" OR "Living Conditions" OR "Social Determinants of Health" OR "Prejudice" OR "Social Stigma" OR "Social Class" OR "Social Marginalization" OR "Occupational Health" OR "Job Insecurity" OR "Unemployment" OR "Food Supply" OR "Medication Adherence" OR "Delayed Diagnosis" OR "Patient Dropouts" OR "Health Services Needs and Demand" OR "Transportation" OR "Rural Health Services")		
LILACS <br>***Search conducted on March 6, 2025***	("Trypanosoma cruzi" OR "Chagas Disease" OR "Neglected Tropical Diseases" OR "Vector-Borne Diseases") AND ("Health Expenditures" OR "Poverty" OR "Cost of Illness" OR "Financial Stress" OR "Health Care Costs") AND ("Quality of Life" OR "Health Status" OR "Mental Health" OR "Depression" OR "Anxiety" OR "Stress, Psychological" OR "Social Support" OR "Social Isolation" OR "Health Services Accessibility" OR "Healthcare Disparities" OR "Insurance, Health" OR "Counseling" OR "Housing" OR "Ill-Housed Persons" OR "Living Conditions" OR "Social Determinants of Health" OR "Prejudice" OR "Social Stigma" OR "Social Class" OR "Social Marginalization" OR "Occupational Health" OR "Job Insecurity" OR "Unemployment" OR "Food Supply" OR "Medication Adherence" OR "Delayed Diagnosis" OR "Patient Dropouts" OR "Health Services Needs and Demand" OR "Transportation" OR "Rural Health Services")	Last 10 years	***39***
BDENF <br>***Search conducted on March 6, 2025***	("Trypanosoma cruzi" OR "Chagas Disease" OR "Neglected Tropical Diseases" OR "Vector-Borne Diseases") AND ("Health Expenditures" OR "Poverty" OR "Cost of Illness" OR "Financial Stress" OR "Health Care Costs") AND ("Quality of Life" OR "Health Status" OR "Mental Health" OR "Depression" OR "Anxiety" OR "Stress, Psychological" OR "Social Support" OR "Social Isolation" OR "Health Services Accessibility" OR "Healthcare Disparities" OR "Insurance, Health" OR "Counseling" OR "Housing" OR "Ill-Housed Persons" OR "Living Conditions" OR "Social Determinants of Health" OR "Prejudice" OR "Social Stigma" OR "Social Class" OR "Social Marginalization" OR "Occupational Health" OR "Job Insecurity" OR "Unemployment" OR "Food Supply" OR "Medication Adherence" OR "Delayed Diagnosis" OR "Patient Dropouts" OR "Health Services Needs and Demand" OR "Transportation" OR "Rural Health Services")	Last 10 years	***4***
Embase <br>***Search conducted on March 6, 2025***	("Chagas disease" OR "Chagas cardiomyopathy") AND (financial stress OR financial crisis OR financial distress OR economic crisis OR economic instability OR economic recession* OR socioeconomic background*) AND ("quality of life" OR "health related quality of life" OR "life quality" OR "quality of life" OR "mental health" OR "condition, mental" OR "health, mental" OR "mental care" OR "mental condition" OR "mental factor" OR "mental help" OR "mental service" OR "mental state" OR "mental status" OR "mental status schedule" OR "psychic health" OR "mental health" OR depression OR "clinical depression" OR "depressive disease" OR "depressive disorder" OR "depressive episode" OR "depressive illness" OR "depressive personality disorder" OR "depressive state" OR "depressive symptom" OR "depressive syndrome" OR depressivity OR "mental depression" OR "parental depression"	Last 10 years	***9***

	OR anxiety OR “isolation stress” OR “isolation, social” OR “psychosocial deprivation” OR “social deprivation” OR “social isolate” OR solitude OR “stress, isolation” OR “social isolation” OR “prejudiced beliefs” OR prejudice OR stigma OR “social stigmatisation” OR “social stigmatization” OR stigmatisation OR stigmatization OR “social stigma” OR “social marginalization” OR “social exclusion” OR “diagnosis delay” OR “delayed diagnosis” OR “care drop out” OR “care dropout” OR “dropout in inpatients” OR “dropout in outpatients” OR “dropout in patients” OR “outpatient dropout” OR “patient drop out” OR “patient drop outs” OR “patient dropouts” OR “therapy drop out” OR “therapy dropout” OR “treatment drop out” OR “treatment dropout” OR “patient dropout” OR “environment, occupational” OR “environment, work” OR “occupational environment” OR “professional climate” OR “work climate” OR “working condition” OR “working conditions” OR “working environment” OR “workplace condition” OR “workplace conditions” OR “workplace environment” OR “work environment” OR “clinical sequelae” OR complications OR “complicative disease” OR “medical sequelae” OR complication)		
Science.gov<br><br>***Search conducted on April 4, 2025***	("Trypanosoma cruzi" OR "Chagas Disease" OR "Neglected Tropical Diseases" OR "Vector-Borne Diseases") AND ("Health Expenditures" OR "Poverty" OR "Cost of Illness" OR "Financial Stress" OR "Health Care Costs") AND ("Quality of Life" OR "Health Status" OR "Mental Health" OR "Depression" OR "Anxiety" OR "Stress, Psychological" OR "Social Support" OR "Social Isolation" OR "Health Services Accessibility" OR "Healthcare Disparities" OR "Insurance, Health" OR "Counseling" OR "Housing" OR "Ill-Housed Persons" OR "Living Conditions" OR "Social Determinants of Health" OR "Prejudice" OR "Social Stigma" OR "Social Class" OR "Social Marginalization" OR "Occupational Health" OR "Job Insecurity" OR "Unemployment" OR "Food Supply" OR "Medication Adherence" OR "Delayed Diagnosis" OR "Patient Dropouts" OR "Health Services Needs and Demand" OR "Transportation" OR "Rural Health Services")	No filters	***11***
Google Scholar<br><br>***Search conducted on March 30, 2025***	("Trypanosoma cruzi" OR "Chagas Disease" OR "Neglected Tropical Diseases" OR "Vector-Borne Diseases") AND ("Health Expenditures" OR "Poverty" OR "Cost of Illness" OR "Financial Stress" OR "Health Care Costs") AND ("Quality of Life" OR "Health Status" OR "Mental Health" OR "Depression" OR "Anxiety" OR "Stress, Psychological" OR "Social Support" OR "Social Isolation" OR "Health Services Accessibility" OR "Healthcare Disparities" OR "Insurance, Health" OR "Counseling" OR "Housing" OR "Ill-Housed Persons" OR "Living Conditions" OR "Social Determinants of Health" OR "Prejudice" OR "Social Stigma" OR "Social Class" OR "Social Marginalization" OR "Occupational Health" OR "Job Insecurity" OR "Unemployment" OR "Food Supply" OR "Medication Adherence" OR "Delayed Diagnosis" OR "Patient Dropouts" OR "Health Services Needs and Demand" OR "Transportation" OR "Rural Health Services")	No filters	***134***
National ETD Portal<br><br>***Search***	("Trypanosoma cruzi" OR "Chagas Disease" OR "Neglected Tropical Diseases" OR "Vector-Borne Diseases") AND ("Health Expenditures" OR "Poverty" OR "Cost of Illness" OR "Financial Stress" OR "Health Care Costs") AND ("Quality of Life" OR "Health Status" OR "Mental Health" OR "Depression" OR "Anxiety" OR "Stress, Psychological" OR "Social	No filters	***5***

conducted on April 9, 2025	Support" OR "Social Isolation" OR "Health Services Accessibility" OR "Healthcare Disparities" OR "Insurance, Health" OR "Counseling" OR "Housing" OR "Ill-Housed Persons" OR "Living Conditions" OR "Social Determinants of Health" OR "Prejudice" OR "Social Stigma" OR "Social Class" OR "Social Marginalization" OR "Occupational Health" OR "Job Insecurity" OR "Unemployment" OR "Food Supply" OR "Medication Adherence" OR "Delayed Diagnosis" OR "Patient Dropouts" OR "Health Services Needs and Demand" OR "Transportation" OR "Rural Health Services")		
CAPES theses and dissertations <br>***Search conducted on March 30, 2025***	("Doença de Chagas" AND "Financeiro")	No filters	***30***
WorldCat <br>***Search conducted on April 7, 2025***	("Trypanosoma cruzi" OR "Chagas Disease") AND ("Poverty" OR "Financial Stress" OR "Health Care Costs") AND ("Quality of Life" OR "Health Services Needs Demand")	No filters	***9***
TOTAL			***554***
