# Supplementary material for: Financial toxicity and the economic burden of Chagas disease: a scoping review
Source: Rev Soc Bras Med Trop. 2026 Oct 16;59:e0233-2026. doi: 10.1590/0037-8682-0233-2026 (PMC13674208; doi:10.1590/0037-8682-0233-2026)
Supplement: Supplementary file 3 [file 1678-9849-rsbmt-59-e0233-2026-md3.pdf]

Supplementary Material 3. *Included studies and reports data.*

STUDY IDENTIFICATION DATA	TARGET POPULATION	AIM	DESIGN	RESULTS
**Financial toxicity related to Chagas disease**				
We have already heard that the treatment doesn't do anything, so why should we take it?": A mixed method perspective on Chagas disease knowledge, attitudes, prevention, and treatment behaviour in the Bolivian Chaco Parisi S, *et al.* *(2020)*	N = 669 people over 18 years and permanent residence in the municipality of Monteagudo (minimum of six months prior to the study).	Additional factors relevant for sustained control as well as protective behaviours within communities should be identified, and valuable lessons learned for future projects.	Mixed method	1. Several barriers were identified, and traditional medicine was often considered more accessible, cheaper, and acceptable within the cultural context. Extreme poverty was associated with CD and the lack of mobility of the rural population, hindering access to information and healthcare.<br>2. Poverty was associated with prioritizing the most urgent issues (work and food) and becoming an easy target for irresponsible private doctors advertising miracle cures.<br>3. Political determinants included the need to prioritize CD as a root societal problem to ensure financial sustainability and the perceived unequal distribution of benefits.
Chagas' cardiomyopathy: the economic burden of an expensive and neglected disease. Abuhab A, *et al*. (2013)	Adult patients with cardiomyopathy of Chagasic (n=58) and non-Chagasic (n=519) origin admitted to a high-complexity university cardiology hospital in the city of São Paulo, Brazil.	To assess the cost burden of CC during acute decompensated HF (ADHF) admissions, and compare this cost to the other etiologies of HF.	Prospective cohort	1. Costs between the groups [Chagasic Cardiomyopathy (CC) and Non-Chagasic (NC)] were compared taking into account different hospital expenses. Patients with CC had higher total costs during admissions. The median room expenses (which also included all indirect costs) were higher in the CC group, but the main difference between the groups was observed when the cost per day was considered.<br>2. Along with the information presented, it is clear that Chagasic patients required more intensive care, which was mainly reflected in the intensive care unit costs included in the room and food section, and drove the higher costs in the CC group.
Analysis of the costs incurred by patients with Chagas disease: The experience in endemic municipalities in Colombia Herazo R, *et al*. (2023)	N = 91 patients with a confirmed diagnosis of Trypanosoma cruzi infection, treated in two endemic municipalities in Colombia.	To understand from the patients' perspective the costs they incur in gaining access to diagnosis and etiological treatment of T. cruzi infection/Chagas disease.	Cross-sectional	1. **Location, time, and costs related to healthcare:** Analysis of the costs and travel time for patients with Chagas disease revealed that treatment at specialized referral hospitals generates higher costs and longer transport times compared to local primary care hospitals. While 58% of patients who went to the local hospital spent less than an hour traveling and 33% had no transportation costs, 62.5% of those treated at referral hospitals spent more than 40,000 COP (US$10.83) on transportation, and 56.2% had similar expenses for accommodation and food. The average cost of transportation to the local hospital was 12,986 COP (US$ 3.52) and to the referral hospital, 68,453 COP (US$ 18.53); the average cost of food and accommodation was 10,626 COP (US$ 2.88) at the local hospital and 57,991 COP (US$ 15.70) at the specialized hospital ($p<0.001$). Despite the high costs of specialized care, only 7.7% of patients reported paying for tests, and two paid for antiparasitic medication (average of 86,500 COP, or US$ 23.42), indicating good coverage by the RIAS benefits plan. Most patients underwent laboratory tests and electrocardiograms at the local hospital, with 92.3% of these being free of charge.

2. **Income Loss (Money Not Earned):** Approximately 46.1% of patients reported income loss when attending appointments at local hospitals and 51.6% at specialized hospitals, with the average loss being significantly higher in specialized hospitals (45,400 COP vs. 22,982 COP). Loss ranged from 10,000 to 39,999 COP. Local care reduces direct costs and income loss, being more economical for the health system, especially in early cases of Chagas disease, avoiding complex treatments. Even so, 70% of patients were referred to specialized hospitals, facing long journeys (more than 4 hours for 67%), mostly by bus (83%), with average transportation costs of 68,453 COP and longer stays.

Patients with Chagas disease treated in referral hospitals experienced significantly higher costs for food, lodging, transportation, and lost income, as well as longer travel times, compared to those treated in primary care units. In addition to these economic and logistical impacts, the study revealed negative effects on patients' quality of life, such as stigmatization, emotional distress, physical limitations affecting work and daily activities, fear of the future, and apprehension regarding available treatments. Decentralizing care to primary care would reduce these costs, facilitate access to treatment, and offer closer support to the patient, promoting greater therapeutic adherence and a more welcoming environment.

STUDY IDENTIFICATION DATA	TARGET POPULATION	AIM	DESIGN	RESULTS
Impact of Socioeconomic Factors on the Survival of Patients on the Waiting List and After Heart Transplantation<br><br>Parra AV,<br><br>(2007)	N = 70 patients who underwent socioeconomic evaluation prior to heart transplantation at the Hospital de Base de São José do Rio Preto between 2000 and November 2005.	Identifying the impact of socioeconomic status on survival of patients listed for heart transplantation as well as in cardiac transplant recipients.	Retrospective longitudinal cohort study	1. Sociodemographic characteristics: Twenty-seven (87%) patients with Chagas disease belonged to the low socioeconomic class, while 33 (85%) patients with other types of cardiomyopathy belonged to this socioeconomic class (p>0.05). Only 4 (13%) patients with Chagas disease and 6 (15%) patients with other cardiomyopathies belonged to the middle socioeconomic class (p > 0.05). On the heart transplant waiting list, 24 (77%) patients with education up to elementary school had Chagas disease, while 29 (74%) patients with other types of cardiomyopathy had completed elementary school (p >0.05). Seven (23%) patients with secondary and/or higher education had Chagas disease, while 10 (26%) patients with other types of cardiomyopathy had secondary and/or higher education (p >0.05). Twenty-nine (94%) of patients with Chagas disease and 37 (95%) of patients without this disease had a caregiver on the heart transplant waiting list (p > 0.05). Twelve (39%) patients receiving retirement benefits had Chagas disease, while 17 (44%) patients receiving this benefit had other types of cardiomyopathy (p > 0.05). Eighteen (58%) patients on the heart transplant waiting list who did not receive financial assistance had Chagas disease and 30 (77%) had another type of cardiomyopathy (p > 0.05).<br><br>2. Waiting time on the transplant list: The median follow-up time for survivors of the surgical procedure was 674 (640) days.Forty-four patients (63%) underwent heart transplantation, 21 (30%) died while on the transplant waiting list, and 5 (7%) were still alive on the waiting list for heart transplantation at the end of the study. Overall, the probability of survival of transplanted patients in the study population 30, 165, 353, and 691 days after transplantation was 88%, 74%, 68%, and 62%, respectively.<br><br>3. Sociodemographic characteristics of transplant recipients: Sixteen (36%) patients who underwent heart transplantation had positive serology for Chagas disease, while 28 (64%) had negative serology (p >0.05). Ten (62%) of the Chagasic patients were male, while 21 (75%) of the non-Chagasic patients were male (p >0.05); the mean age was 43 ± 10 years in Chagasic patients and 44 ± 12 in non-Chagasic individuals (p >0.05), while the mean weight was 56 ± 9 kg in Chagasic patients and 69 ± 16 kg in non-Chagasic patients (p > 0.05). The median monthly income was 2.5 (2.75) minimum wages in individuals with Chagas disease, and 4 (4) minimum wages in individuals without this disease (p >0.05). The median distance from residence to the city of São José do Rio Preto was 90 (145) km in Chagasic patients and 100 (230) km in non-Chagasic patients (p >0.05).<br><br>4. Post-transplant survival: The probability of survival 16, 123, and 598 days after heart transplantation for Chagas disease patients was 69%, 62%, and 53%, respectively, while the probability of survival for non-Chagas disease patients 19, 158, and 575 days after transplantation was 85%, 81%, and 71%, respectively.

Funding for Chagas Disease: A 10-Year (2009–2018) Survey.<br>Sangenito LS, Branquinha MH, Santos ALS<br><br>(2020)	Annual funding data on neglected diseases provided by hundreds of institutions from the government, private, and philanthropic sectors.	To evaluate the impact data of financial investment applied to Chagas disease compared to other neglected diseases.	Epidemiological study using secondary data.	1. This paper summarized the investments applied to Chagas disease over a 10-year period. As we have seen, little funding was directed to this specific parasitic disease compared to other neglected diseases. For a human disease responsible annually for approximately 700,000 DALYs, with an economic impact of over US$7 billion, receiving only 0.67% (US$236.3 million) of the total budget for neglected diseases (considering the period from 2009 to 2018) is unacceptable and meaningless.<br><br>2. Therefore, allocating more resources to combat Chagas disease is urgently needed in terms of saving lives, restoring quality of life, and reducing the economic impact on health systems.<br><br>3. Regarding Chagas disease, it is clear that funding depends much more on the public sector (74%) than on other areas (private and philanthropic). However, public agencies in Latin American countries contribute very little compared to the NIH-USA, a situation that needs to be reviewed.<br><br>4. Furthermore, development agencies, governments, and the Chagas disease community must work together to ensure that (scarce) resources are well applied. In parallel, there is a need to adopt market strategies and incentives to attract more pharmaceutical industry involvement in the fight against Chagas disease, given that the sector contributed only US$44.4 million over 10 years.<br><br>5. Other relevant issues to be addressed include the low investment in important tools such as diagnostics, vaccines, and vector control, which together received only 10% of the total budget, with the remainder equally distributed between basic research and drug development/repositioning.
Metabolic, mental health, behavioural and socioeconomic characteristics of migrants with Chagas disease in a non-endemic country.<br>Jackson Y, *et al*.<br><br>(2012)	N = 137 Latin American migrants over 15 years old with Chagas disease at the University Hospitals of Geneva, Switzerland.	To evaluate the presence of cumulative metabolic, mental health, behavioural and socioeconomic factors in a cohort of Latin American migrants with Chagas disease in Geneva, Switzerland.	Cross-sectional study	Specific socioeconomic findings highlighting the financial burden include:<br><br>1. Widespread poverty: The vast majority of participants (89.1%) lived below the Swiss poverty line.<br><br>2. Precarious immigration status: 83.3% of participants did not have legal residency permits (were "undocumented").<br><br>3. Lack of access to health insurance: The majority (72.3%) did not have health insurance.<br><br>4. Barriers to access to care: The study concludes that the lack of residency permits and the lack of health insurance prevented most participants from accessing the Swiss healthcare system through mandatory insurance.<br><br>5. Working conditions: The majority of participants (81.2%) worked as domestic employees. Furthermore, 34.9% reported that their overall social situation in Switzerland was worse than before migration.<br><br>6. Economic burden and health: The article points out that these factors, including "heavy economic burden," poor housing and working conditions, and social exclusion, are known to combine to increase

				morbidity and mortality.<br><br>7. Communication barriers: In addition to financial factors, 58.4% of participants reported difficulties in adequate communication with healthcare professionals, representing an additional barrier to care.
The Cost of Lost Productivity Due to Premature Chagas Disease-Related Mortality: Lessons from Colombia (2010–2017).<br>Olivera MJ, Palencia-Sánchez F, Riaño-Casallas M<br><br>(2021)	N = 1261 deaths from Chagas disease in 2010-2017 among men aged 18 to 62 or women aged 57 in Colombia	To estimate the PYWL associated with premature deaths caused by Chagas disease during the period from 2010 to 2017 in Colombia.	Epidemiological study using secondary data.	1. In total, 1,261 deaths were analyzed in the study, of which 60% corresponded to men. The average age at death was 21 years. The post-traumatic mortality rate (PTMR) was lower in women than in men (18,384 vs. 30,237), and the total Potential Years of Work Lost was 48,621.<br><br>2. The cost of Years of Life Lost, premature mortality, for both sexes combined was US$29,683,913 during the study period, and was US$17.3 million for men and US$12.3 million for women from 2010 to 2017. In the case of women, they tended to have lower wages and a shorter working life.<br><br>3. The total cost of lost productivity due to premature mortality was 39.7% higher in men than in women, although the cost per Years of Work Lost was higher in women.
Economic costs of Chagas disease in Colombia in 2017: a social perspective. Oliveira MJ, Buitrago G<br><br>*(2020)*	N = 548 and direct medical, non-medical, and indirect care costs associated with patients with Chagas disease.	Quantifying the various components of the costs of Chagas disease from a social perspective in Colombia for the year 2017.	Cross-sectional study using secondary data.	1. **Direct medical costs attributed to Chagas disease:** Direct medical expenses associated with Chagas disease in Colombia in 2017 totaled US$3,059,000 directly attributed to the disease and US$5,751,388 as the total cost for diagnosed individuals. The largest expenses attributed to Chagas were for the implantation of cardiac devices (27%), tests to assess ventricular function (13%), and myocardial perfusion diagnostic techniques (13%). Other relevant costs included medical consultations, rehabilitation, hospitalization, and emergency care.<br><br>2. **Direct non-medical costs attributed to Chagas disease:** Direct non-medical expenses totaled US$1,471,676 in 2017. The main cost components were transportation (33%), laboratory tests (20%), and food (17%). Accommodation accounted for 15% of the total, followed by medical fees (9%), cardiological diagnostic tests (4%) and hospital co-payments (2%), highlighting the extra-hospital financial impact suffered by patients with Chagas disease.<br><br>3. **Indirect costs attributed to Chagas disease:** Indirect expenses related to lost productivity reached US$ 5,881,571 in 2017. Presenteeism was the biggest contributor, with 109 million lost days and 53% of the total cost, followed by absenteeism with 83 million days (38%) and mortality, with 213 deaths and a 9% impact. These data highlight the high economic cost of the disease even outside the clinical setting.<br><br>4. **Estimated cost of the disease for Chagas disease:** The estimated national cost of Chagas disease in Colombia in 2017 was US$ 13.1 million, of which US$ 7.2 million (55.1%) represented direct expenses for medical care associated with the disease (only 1.2% of the total estimated population) and US$ 5.8 million (44.9%) represented lost productivity due to absenteeism, reduced work productivity, and premature mortality. The total annual cost to society of a person with Chagas disease was US$4,226.

Chagas disease and perceived quality of life: a cross-sectional study. Baldoni NR, et al.<br><br>*(2023)*	N = 611 (328 with CD and 283 without CD) participants from two blood donation centers: São Paulo (capital) and Montes Claros, Minas Gerais, Brazil. Participants with CD were identified in blood donations by serological diagnosis between 2008 and 2010, and those without CD were donors with negative serology identified during the same period.	To assess and compare QoL between individuals with and without ChD.	This cross-sectional study was conducted within a concurrent cohort study (REDS).	1. **Educational level:** Among all participants, the majority (51.8% with CDh and 50.9% without CDh) were women. The mean age for participants with and without CDh was 56.2 (±9.9) years and 58.4 (±9.6) years, respectively. The mean monthly income was US$22.97 (±15.49) vs. US$32.07 (±22.37) (p<0.001). Furthermore, the educational level and the percentage of participants who "can read and write" (79.6% vs. 92.6%, p<0.001) were lower in participants with CDh. The majority of participants in both groups were Black/mixed race (64.4% vs. 54.1%, p=0.034).<br><br>2. **Sociodemographic, behavioral, and clinical characteristics of participants with and without Chagas disease:** The majority in both groups did not engage in physical activity (54.6% with CD and 52.7% without CD) nor consume alcohol in the days prior to answering the questionnaire (65.6% and 57.4%, respectively). Furthermore, cardiomyopathy was significantly (p<0.001) more frequent in participants with CD (29.1% vs. 4.9%), as were significant electrocardiographic abnormalities (39.6% vs. 22.6%; p<0.001). The main comorbidities among both groups were systemic arterial hypertension (39.8% and 36.9%, respectively) and diabetes mellitus (13.1% and 13.5%, respectively) (Table 1). Polypharmacy use (use of ≥5 medications) was 2.5% and 2.1%, respectively.<br><br>3. **Quality of life:** The results of the quality of life assessment are shown in Table 2. All QoL domains of participants with CDh showed lower averages when compared to those without CDh. The most compromised QoL domain was the environment in both groups, with averages of 64.52 and 66.8 for participants with and without CDh, respectively. For all OR values < 1, participants with CDh had lower QoL than those without CDh (reference). Without controlling for confounding factors, a significant difference was observed in three of the four domains assessed: physical (p = 0.001), psychological (p = 0.001), and environmental (p = 0.010). In the multivariate models, after multiple imputations, statistically significant differences were observed in the physical (p = 0.018) and psychological (p = 0.007) domains. Figure 2 shows the violin plots of the distribution of quality of life by domain between the two groups.

STUDY IDENTIFICATION DATA	TARGET POPULATION	AIM	DESIGN	RESULTS
Opportunity cost for early treatment of Chagas disease in Mexico<br><br>Ramsey JM, *et al*.<br><br>(2014)	Patients with Chagas disease in Mexico (simulated cohort from birth to death).	To estimate the current costs of treating a chronic CD case detected and treated early vs an undetected case among the salaried population (47% of the Mexican population , and the direct and indirect costs and effects simulated since birth to death using a cohort Markov model.	Economic modeling study	The total lifetime cost per patient is lower when there is early diagnosis and treatment (US$10,160), increasing to US$11,558 when only 80% receive treatment, and reaching US$11,877 when no patient is diagnosed or treated. The largest source of financial toxicity identified was indirect costs: loss of productivity, which represented 44% of costs in scenarios with treatment and up to 75% in cases without diagnosis, demonstrating the economic impact of untreated disease. Direct costs included hospitalizations, medical consultations, laboratory tests, imaging, and medications, and were proportionally lower, representing approximately 25% to 56% of the total, depending on the scenario. The cost of treatment increases 24 times from the acute phase to the indeterminate phase, and the cost of the chronic phase for untreated patients is almost double the cost for those who receive treatment. Therefore, the study concludes that early diagnosis and treatment are cheaper in the long run, reduce direct costs, and, most importantly, avoid economic losses related to productivity, which are the main drivers of financial toxicity.
Inequalities in the social determinants of health and Chagas disease transmission risk in indigenous and creole households in the Argentine Chaco.<br><br>Fernández MDP, Gaspe MS, Gürtler RE<br><br>(2019)	7 communities and 587 rural homes in the municipality of Pampa del Indio	The gap in our understanding of the combined effects of structural and intermediary SDHs on key vector indices closely associated with the risk of vector-borne transmission of T. cruzi	Quantitative, observational study	The demographic and socioeconomic characteristics of Qom households underperformed those of Creole households at both baseline (2008) and during the 2012-2015 period (Table 2). Creole households inhabited larger, higher-quality houses with less available shelter (Fisher's exact test, $P < 0.001$). Qom housing quality improved significantly between 2008 and 2012-2015 (Fisher's exact test, $P < 0.001$). Qom households had greater overcrowding and lower educational levels than Creole households (Fisher's exact test, $P < 0.001$ and $P = 0.03$, respectively). In general, formal employment was scarce, and the main economic activities and sources of income were related to agricultural and animal husbandry practices for both ethnic groups. A significantly larger proportion of Creole families based their livelihoods on agricultural or animal husbandry practices (Fisher's exact test, $P = 0.03$) and had higher goat equivalence indices than Qom families (Fisher's exact test, $P = 0.01$), who were more dependent on social support (Fisher's exact test, $P = 0.01$).
Quality of life and associated factors in patients with chronic Chagas disease<br>Santos-Filho JCL, *et al.*<br><br>*(2018)*	N = 361 outpatients followed at the Evandro Chagas National Institute of Infectious Diseases, Brazil	To evaluate the quality of life (QoL) of patients with Chagas disease (CD) and the association between QoL domains and several clinical, socioeconomic and lifestyle characteristics of this population.	Cross-sectional observational study	Univariate analysis demonstrated a significant association between female sex, education level, race, clinical presentation of CD, obesity, smoking, alcohol consumption, physical activity level, functional class, per capita income, and sleep duration with the physical domain. Age, sex, education level, race, clinical presentation of CD, systemic arterial hypertension, obesity, alcohol consumption, physical activity level, functional class, number of people living in the household, per capita income, and sleep duration were associated with the psychological domain. The variables education level, smoking, alcohol consumption, physical activity level, functional class, number of people living in the household, and clinical presentation of CD showed an association with the social relationship domain. Sex, education level, race, clinical presentation of CD, smoking, level of physical activity, functional class, number of people living in the household, per capita income, and sleep duration were associated with the environment domain. The variables that showed an association with the overall quality of life domain in the univariate analysis were sex, education level, clinical presentation of CD, obesity, alcohol consumption, level of physical activity, functional class, number of people living in the household, and per capita income.

STUDY IDENTIFICATION DATA	TARGET POPULATION	AIM	DESIGN	RESULTS
Barriers to Diagnosis Access for Chagas Disease in Colombia.<br><br>Oliveira MJ, *et al*.<br><br>(2018)	N = 16 key informants, who were health policymakers, department heads, hospital directors, and primary care physicians with knowledge and experience in treating cases of Chagas disease.	Identify potential barriers to healthcare access for Chagas disease in Colombia, defined as diagnosis, from the perspective of healthcare providers, and, additionally, to systematically review the literature on the views of healthcare professionals to the access to healthcare for Chagas disease and to identify potential barriers.	Qualitative study	Funding and Payment. The results of the interviews suggest that funding is a fundamental barrier limiting access to diagnosis for many patients with Chagas disease. In Colombia, tests for the diagnosis of T. cruzi in both the acute phase (direct methods) and the chronic phase (indirect methods) are part of the basic services package of the mandatory health plan, and anyone with subsidized health insurance (without the ability to pay) or a contributory scheme (with the ability to pay) can access them. Despite this, most patients have to bear the cost of approximately US$25 for the confirmatory test (IFA) due to a lack of laboratories in primary care centers that perform this second test. Furthermore, patients must pay a co-payment for each medical consultation and each test (a percentage payment that must be made by beneficiaries of the contributory scheme and all affiliates of the subsidized scheme, approximately US$1 to US$8). Alternatively, they must pay a co-payment (a cash contribution to be paid by all members of the contributory plan, approximately US$1 to US$8). In addition, patients must bear the costs of transportation to primary care centers (~US$3) and travel to laboratories (~US$3 to US$25), usually located in cities. On the other hand, coverage rates for health plans are less than 100%, especially in rural areas. People without health insurance face greater barriers to care, delay seeking care, and have more unmet needs.
Global economic burden of Chagas disease: a computational simulation model.<br>Lee BY, et al.<br><br>(2013)	We used TreeAge Pro (version 2009) and Microsoft Excel (version 2007) to develop a computational Markov simulation model, adapted from reported models.	To develop a simulation model to estimate the global health and economic burden of Chagas disease from the societal perspective (a commonly used economic perspective that includes health-care costs plus cost related to work absenteeism).	Quantitative, observational study	**Results:** On average, an infected individual incurs US$474 in healthcare costs and 0.51 DALYs annually. Over their lifetime, an infected individual accumulates an average net present value of US$3,456 and 3.57 DALYs. Globally, the annual burden is US$627.46 million in healthcare costs and 806,170 DALYs. The global net present value of currently infected individuals is US$24.73 billion in healthcare costs and 29,385,250 DALYs. Converting this burden into costs results in annual costs per person of US$4,660 and lifetime costs per person of US$27,684. Global costs are US$7.19 billion per year and US$188.80 billion per lifetime. More than 10% of these costs emanate from the US and Canada, where Chagas disease is not traditionally endemic. A substantial proportion of the burden arises from lost productivity due to premature mortality induced by cardiovascular diseases.<br><br>**Interpretation:** The economic burden of Chagas disease is similar to or exceeds that of other major diseases worldwide (e.g., rotavirus US$2.0 billion, cervical cancer US$4.7 billion), even in the US (Lyme disease US$2.5 billion), where Chagas disease is not traditionally endemic, suggesting an economic argument for more attention and efforts toward controlling Chagas disease.
