# Supplementary material for: Financial toxicity and the economic burden of Chagas disease: a scoping review
Source: Rev Soc Bras Med Trop. 2026 Oct 16;59:e0233-2026. doi: 10.1590/0037-8682-0233-2026 (PMC13674208; doi:10.1590/0037-8682-0233-2026)
Supplement: Supplementary file 4 [file 1678-9849-rsbmt-59-e0233-2026-md4.pdf]

Supplementary Material 4. Original articles excluded from the full-text review.

Nº	TITTLE	AUTHOR	EXCLUSION REASON
1	Home improvement and system-based health promotion for sustainable prevention of Chagas disease: A qualitative study	NIETO-SANCHEZ, Claudia *et al*.	Does not fit the exposure criteria
2	Enfermedad de Chagas en México	SALAZAR-SCHETTINO, Paz María *et al*.	Does not fit the exposure criteria
3	Cardiac global risk in latin American migrants with Chagas disease: Metabolic factors, socio-demographic characteristics and mental health status	JACKSON, Y. *et al*. (2011)	Does not fit the outcome criteria
4	Making Sense of Chagas Disease among Mexican Immigrants in California	VALDEZ TAH, Alba Rocío.	Does not fit the outcome criteria
5	Neglected tropical diseases: A biosocial perspective	GALHOTRA, Abhiruchi; MISHRA, Abhisek.	Does not fit the exposure criteria
6	Comparison of Anxiety and Depression Symptoms in Individuals According to their Sex, Type of Cardiac Device, and Diagnosis of Chagas Disease	DESSOTTE, Carina Aparecida Marosti *et al*.	Does not fit the exposure criteria
7	Anxiety, depression, and memory loss in Chagas disease: a puzzle far beyond neuroinflammation to be unpicked and solved	LANNES-VIEIRA, Joseli *et al*.	Does not fit the source criteria
8	Quality of life and depressive symptoms in Chagas disease patients	Ozaki, Guariento and De Almeida	Does not fit the source criteria
9	The Association Between Physical Performance and Health-Related Quality of Life Based on the EuroQol 5-Dimensional Questionnaire in Patients With Chagas Disease	Lins, Tura and Kasal	Does not fit the source and exposure criteria
10	Quality of life and associated factors in patients with chronic Chagas disease	Santos-Filho, Vieira, Xavier *et al*.	Does not fit the source and exposure criteria
11	Qualidade de vida de adultos e idosos portadores da Doença de Chagas	Dias	Does not fit the exposure criteria
12	Determinantes sociais da saúde e mortalidade por doenças negligenciadas no Brasil: uma abordagem espacial e temporal	Melo	Does not fit the source criteria
13	Explanatory models and cultural consensus: the case of Chagas disease in Santa Cruz, Bolivia	Forsyth	Does not fit the exposure criteria

Nº	TITTLE	AUTHOR	EXCLUSION REASON
14	A atividade de trabalho dos agentes e gestores do Programa de Controle da Doença de Chagas na região do Vale do Jequitinhonha: uma análise de conteúdo	Queiroz	Does not fit the population
